# Therapeutic effects of intranasal tocotrienol-rich fraction on rhinitis symptoms in platelet-activating factor induced allergic rhinitis

**DOI:** 10.1186/s13223-022-00695-x

**Published:** 2022-06-13

**Authors:** Cheryl Wei Ling Teo, Stephanie Jia Ying Png, Yee Wei Ung, Wei Ney Yap

**Affiliations:** 1 Research and Development Department, Davos Life Science, 3 Biopolis Drive, #04-19, Synapse, 138623 Singapore, Singapore; 2 Research and Development Department, KL-Kepong Oleomas (KLK Oleo), Level 8, Menara KLK, No 1, Jalan PJU 7/6, Mutiara Damansara, 47810 Petaling Jaya, Selangor Malaysia; 3 School of Biological Sciences, Nanyang Technological University, 50 Nanyang Avenue, 639798 Singapore, Singapore

**Keywords:** Allergic rhinitis, Tocotrienol-rich fraction, Tocotrienol, Antioxidant, Anti-inflammation, Anti-allergic, Platelet-activating factor

## Abstract

**Background:**

Platelet-activating factor (PAF) has been suggested to be a potent inflammatory mediator in Allergic rhinitis (AR) pathogenesis. Vitamin E, an essential nutrient that comprises tocopherol and tocotrienol, is known as a potential therapeutic agent for airway allergic inflammation. This study aimed to investigate the beneficial effects of intranasal Tocotrienol-rich fraction (TRF) on PAF-induced AR in a rat model.

**Methods:**

Sprague Dawley rats were randomly assigned into 3 groups: Control, PAF-induced AR and PAF-induced AR with TRF treatment. To induce AR, 50 μl of 16 μg/ml PAF was nasally instilled into each nostril. From day 1 to 7 after AR induction, 10 μl of 16 μg/μl TRF was delivered intranasally to the TRF treatment group. Complete upper skulls were collected for histopathological evaluation on day 8.

**Results:**

The average severity scores of AR were significantly higher in the PAF-induced AR rats compared to both control and PAF-induced AR with TRF treatment. The histologic examination of the nasal structures showed moderate degree of inflammation and polymorphonuclear cells infiltration in the lamina propria, mucosa damage and vascular congestion in the PAF-induced AR rats. TRF was able to ameliorate the AR symptoms by restoring the nasal structures back to normal. H&E staining demonstrated a statistically significant benefit upon TRF treatment, where minimal degree of inflammation, and a reduction in the infiltration of polymorphonuclear cells, mucosa damage and vascular congestion were observed.

**Conclusion:**

TRF exhibited symptomatic relief action in AR potentially due to its antioxidant, anti-inflammatory and anti-allergic properties.

## Introduction

Allergic rhinitis (AR) is a common inflammatory disorder of the nasal mucosa that is clinically symptomized by sneezing, nasal itching, rhinorrhoea, and nasal congestion [1]. It has been suggested as a prevalent yet undertreated respiratory disease affecting up to 30% of adults and 40% of children over the world [2]. AR substantially impacts the socioeconomic burden in terms of increased healthcare costs and decreased productivity, as well as impairment in quality of life, causing public health concerns [3, 4].

The pathophysiology of AR involves a complex Immunoglobulin E (IgE)-mediated hypersensitivity reaction triggered by inhaled allergens. Upon exposure, immune cells process the allergens and stimulate B-cell mediated immune response. The cross-linking of IgE antibody on the surface of the activated inflammatory cells triggers mast cell degranulation in the early phase of allergic response, releasing preformed and newly synthesized mediators such as platelet-activating factor (PAF), histamine and proinflammatory cytokines that promote vascular permeability, rhinorrhoea and vasodilation [5, 6]. These mediators perpetuate the allergic response to the late phase, triggering cellular inflammation. The recruitment of several inflammatory cells, including eosinophils, basophils, monocytes and lymphocytes, continues promoting the release of the mediators and remodel of the nasal tissues. This resulted in nasal congestion, the most common symptom reported by AR patients [7]. PAF is a potent lipid inflammatory mediator released by several cell types [8], whose role was first reported in literature by a French immunologist, Jacques Benveniste, as a mediator of anaphylaxis [9]. In AR, PAF has been recognized to have an important role in inducing vascular permeability associated with rhinorrhoea and nasal congestion, consequently nasal hyperreactivity is then encouraged [10–12]. Furthermore, PAF serves as a powerful chemoattractant for eosinophils and neutrophils to the site of allergic inflammation. PAF induces leukocytes degranulation and adhesion, triggering the production and release of free radicals such as superoxide anion and hydroxyl anions in the nasal mucosa [13]. Overproduction of the free radicals generates oxidative stress (OS) and exacerbates inflammation in the cell. As such, therapeutic antioxidants could potentially be adopted to provide better control of the inflammation in AR and prevent symptoms aggravation.

Vitamin E is a robust lipid soluble antioxidant that presents peroxyl radical-scavenging activity. It comprises of 2 homologs, Tocopherol (TP) and Tocotrienol (T3), with each homolog further classified into α, β, γ and δ isoforms. TP and T3 have the same basic chemical structure characterized by a long chain hydrocarbon at the 2-position of a chromanol ring. TP consists of a side chain made of a phytylin group while T3 consists of an isoprenyl group with 3 unsaturated double bonds at positions 3’,7’ and 11’ [14, 15]. Both TP and T3 display antioxidant properties, where T3 has shown a more potent free radical scavenging effect than TP due to their better penetration and distribution in the lipid layers of the cell membranes [16–18]. Begum et al. concluded a higher incorporating activity of α-T3 than α-TP resulting in an increased protection against erythrocyte oxidation and impairing its deformability [19]. Furthermore, Tocotrienol-rich fraction (TRF) at 50 μM was reported to show a greater protective effect against LDL oxidation than TP in a cell line study [20]. Evidences also indicated T3 to be more superior than TP in anti-inflammation by lowering the proinflammatory mediators and suppressing the proinflammatory signaling such as nuclear factor kappa B (NF-κB) and signal transducer and activator of transcription 3 (STAT3) [21–23].

Currently, therapeutic interventions of AR include pharmacotherapy and immunotherapy [24]. According to the Allergic Rhinitis and Its Impact on Asthma (ARIA) guidelines, oral antihistamines and nasal decongestant are the first line medications for mild to moderate AR. In more serious cases, second line medications such as intranasal corticosteroids or allergen immunotherapy could be considered [25]. However, there are undesired side effects with long term use of these AR treatments. Vitamin E has been known as a potential therapeutic agent for airway allergic inflammation, though TP was dominantly focused in most of the research [26–29]. This study investigated the influence of PAF on the development of AR in an animal model. Subsequently, the efficiency of intranasal administration of palm-derived TRF in alleviating the symptoms of AR and histological changes in the nasal structures were determined. By elucidating the pharmacotherapy benefits of TRF, this study may help to develop TRF as a therapeutic agent in the AR management.

## Methods

### Drugs and chemicals

Tocotrienol-rich fraction (TRF) was obtained from Davos Life Science Sdn. Bhd. (Malaysia). Platelet-activating factor (PAF), bovine serum albumin (BSA), glycerine, tween-80, ketamine chloride, xylazine hydrochloride and pentobarbital were obtained from Sigma-Aldrich (St. Louis, MO, USA).

### Animals

This study was conducted with male Sprague Dawley rats aged 8 to 9 weeks (weighing 250 to 300 g), that were without any evidence of upper airway infection. The rats were purchased from Invivos Pte Ltd and housed at the Biological Resource Centre (BRC), A*Star Singapore under standard laboratory conditions (12 h light/12 h dark schedule at 24 °C ± 2 °C). The rats were fed with standard pellet diet and water ad libitum. This study was approved by the BRC Institutional Animal Care and Use Committee (IACUC).

### Allergic rhinitis (AR) model

The rats were acclimatized to laboratory conditions for 72 h prior to experiment and randomly assigned to 3 groups. Group 1 (n = 5) is the control group where the healthy rats were not exposed to the AR. Group 2 (n = 7) represents the PAF-induced AR group where the rats were intranasally induced with 50 µl of 16 µg/ml PAF per nostril. Group 3 (n = 7) is the PAF-induced AR with TRF treatment group where the AR rats additionally received 10 µl of 16 µg/µl TRF per nostril.

**Fig. 1:**
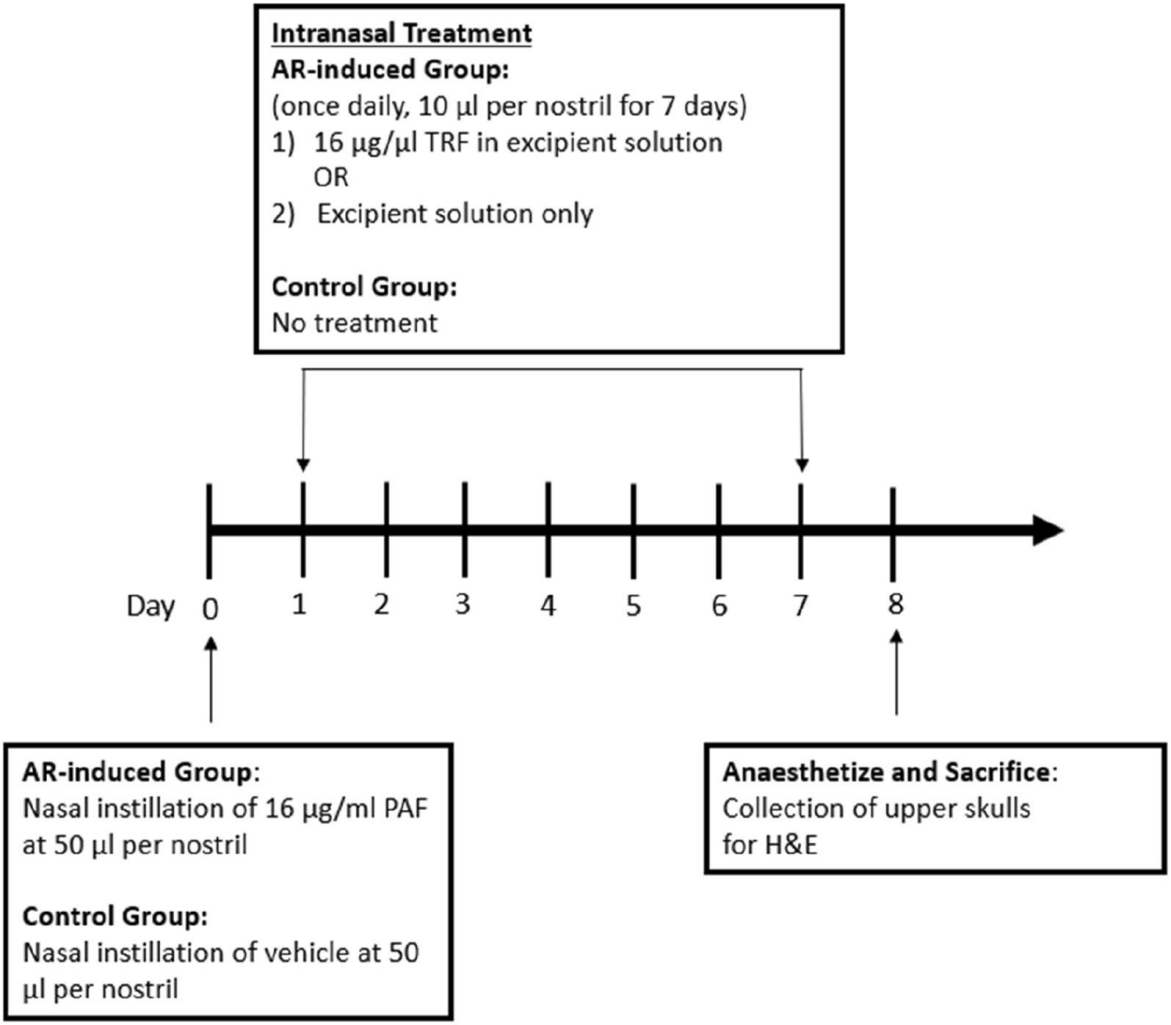
PAF-induced AR animal model. The rats were intranasally instilled with PAF on day 0 to induce AR. Treatment with TRF in excipient solution or pure excipient solution was administered intranasally to the AR rats from day 1 to 7. No treatment was given to the control rats. All the rats were sacrificed on day 8 and upper skulls were collected for histological examination using H&E. AR, Allergic rhinitis; H&E, Hematoxylin and eosin; PAF, Platelet-activating factor; TRF, Tocotrienol-rich fraction

The experimental design for the AR induction and treatment was shown in Fig. 1. Briefly, the rats were subjected to a combination of 75 mg/kg of ketamine chloride and 10 mg/kg of xylazine hydrochloride anaesthesia intraperitoneally. Isoflurane inhalational anaesthetic agent was avoided to prevent irritation of the nasal mucosa. AR was induced via a one-time induction of PAF to the nasal cavity. The PAF stock was prepared in absolute ethanol and subsequently diluted with phosphate-buffered saline containing 0.25% BSA to working concentration. On day 0, 50 µl of 16 µg/ml PAF was instilled into each nostril of the AR-induced rats by placement of small droplets using a micropipette. The same amount of vehicle was applied to the control group.

From day 1 to 7 post-induction, the AR-induced rats were administered with 10 µl of excipient solution containing 5% saline, 5% glycerine, 5% tween-80 and 85% water into each nostril. While the TRF treatment group received additional 16 µg/µl of TRF in the same excipient solution at 10 µl per nostril. No treatment was given to the control group.

### Histology examination

All the rats were euthanized with 120 mg/kg of pentobarbital on day 8. The rats were decapitated and the eyes, skin, fur, muscle tissues, and the mandibles were excised. The complete upper skull was then fixed in 10% buffered formalin. The samples were dehydrated in ascending grades of alcohol and xylene and embedded in paraffin for histological examination by Advanced Molecular Pathology Laboratory (AMPL), A*STAR. Tissue sections were taken from paraffin blocks at 5 µm thickness and stained with hematoxylin and eosin (H&E). The stained samples were evaluated with a light microscope by a certified veterinary pathologist blinded to the protocol.

### Scoring of AR severity

The significance of nonneoplastic lesions in nasal tissues was evaluated semi quantitatively by applying grading scheme of 0 to 5 severity grades with reference to previous reports [30, 31]. Severity grading is considered to be semi quantitative as it relies on the estimation of severity rather than actual measurement.

### Statistical analysis

The data were analysed using Graphpad Prism version 9.1.0 (Graphpad Softare, San Diego, CA). Comparison of the histological evaluation graded by the veterinary pathologist in all groups were performed with one-way ANOVA and subsequently post-hoc Tukey test. Statistical significance value was set at p < 0.05.

## Results

### TRF reduced average severity scores of AR in PAF-induced rats

**Fig. 2:**
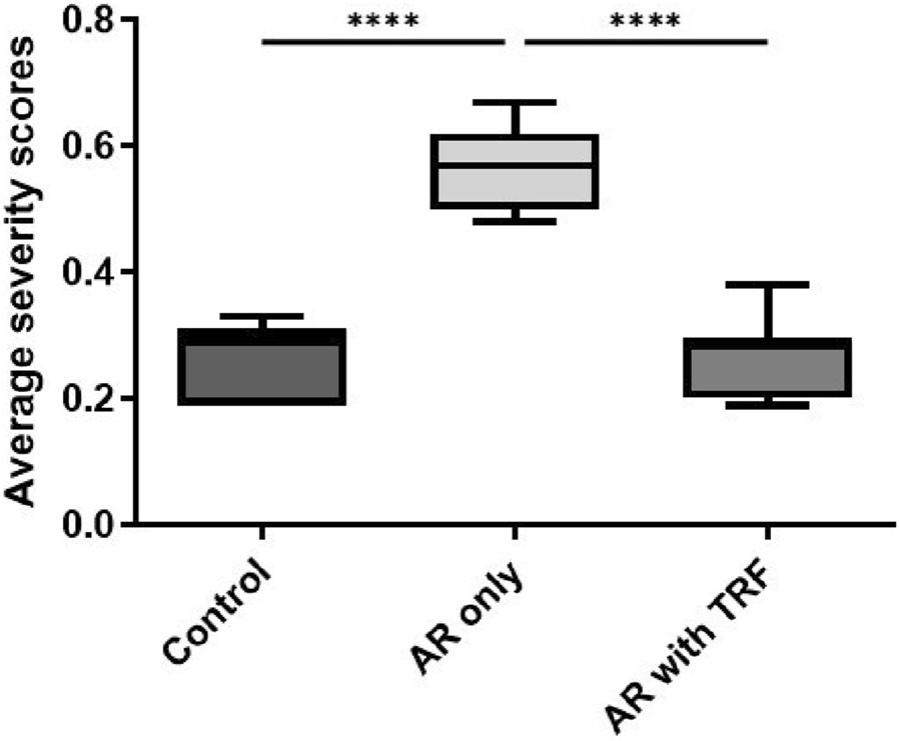
The average severity scores of AR in Control, PAF-induced AR and PAF-induced AR with TRF treatment groups. Data are representative of mean ± standard error (n = 19); ****p < 0.0001. AR, Allergic rhinitis; PAF, Platelet-activating factor; TRF, Tocotrienol-rich fraction

Figure 2 shows the average severity scores of AR in the Control, PAF-induced AR and PAF-induced AR with TRF treatment group. Overall, PAF aggravated the AR symptoms by showing the highest average severity score of 0.56, while TRF was able to mitigate the damage significantly by lowering the severity score to 0.27 that is close to control (0.26).

### TRF showed protective effects against AR symptoms in PAF-induced rats

**Fig. 3:**
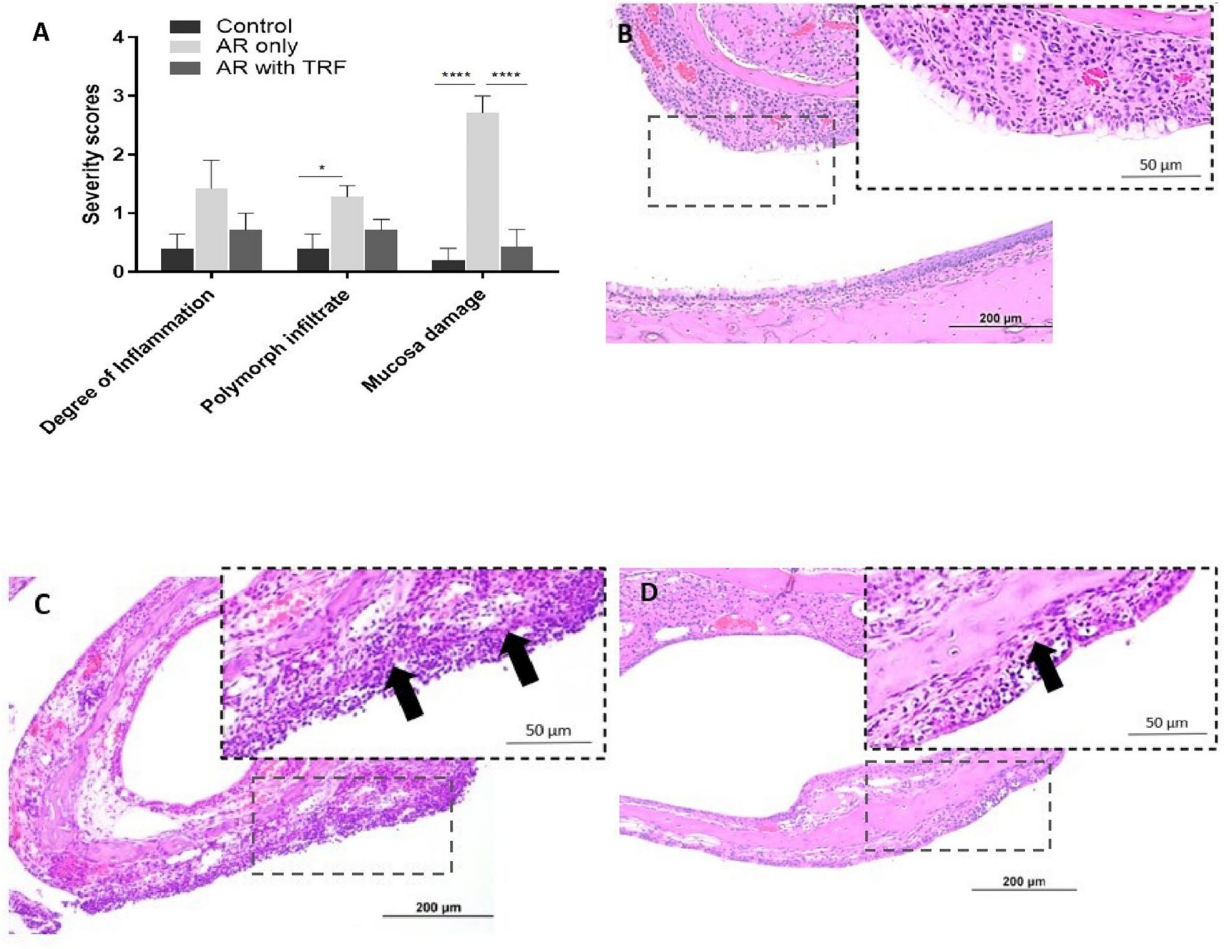
The severity scores of AR for histological evaluation (**A)** and light microscopic images of H&E staining in nasal section (**B**–**D**; ×200 magnification). (**B)** Control group shows regular nasal structures with no inflammation. (**C)** PAF-induced AR group shows moderate inflammation, aggregates of polymorphonuclear cells, thickening of lamina propria and epithelial loss. (**D)** Regular nasal structures with minimal inflammation and aggregates of polymorphonuclear cells were observed in PAF-induced AR with TRF treatment group. Black arrows indicate inflammation and polymorphonuclear cells infiltration in the submucosa. The subjective scoring represents 0: no significant abnormalities; 1: minimal; 2: mild; 3: moderate; 4: marked; 5: severe. Data are representative of mean ± standard error (n = 19); *p < 0.05, ****p < 0.0001. AR, Allergic rhinitis; H&E, Hematoxylin and eosin; PAF, Platelet-activating factor; TRF, Tocotrienol-rich fraction

**Fig. 4:**
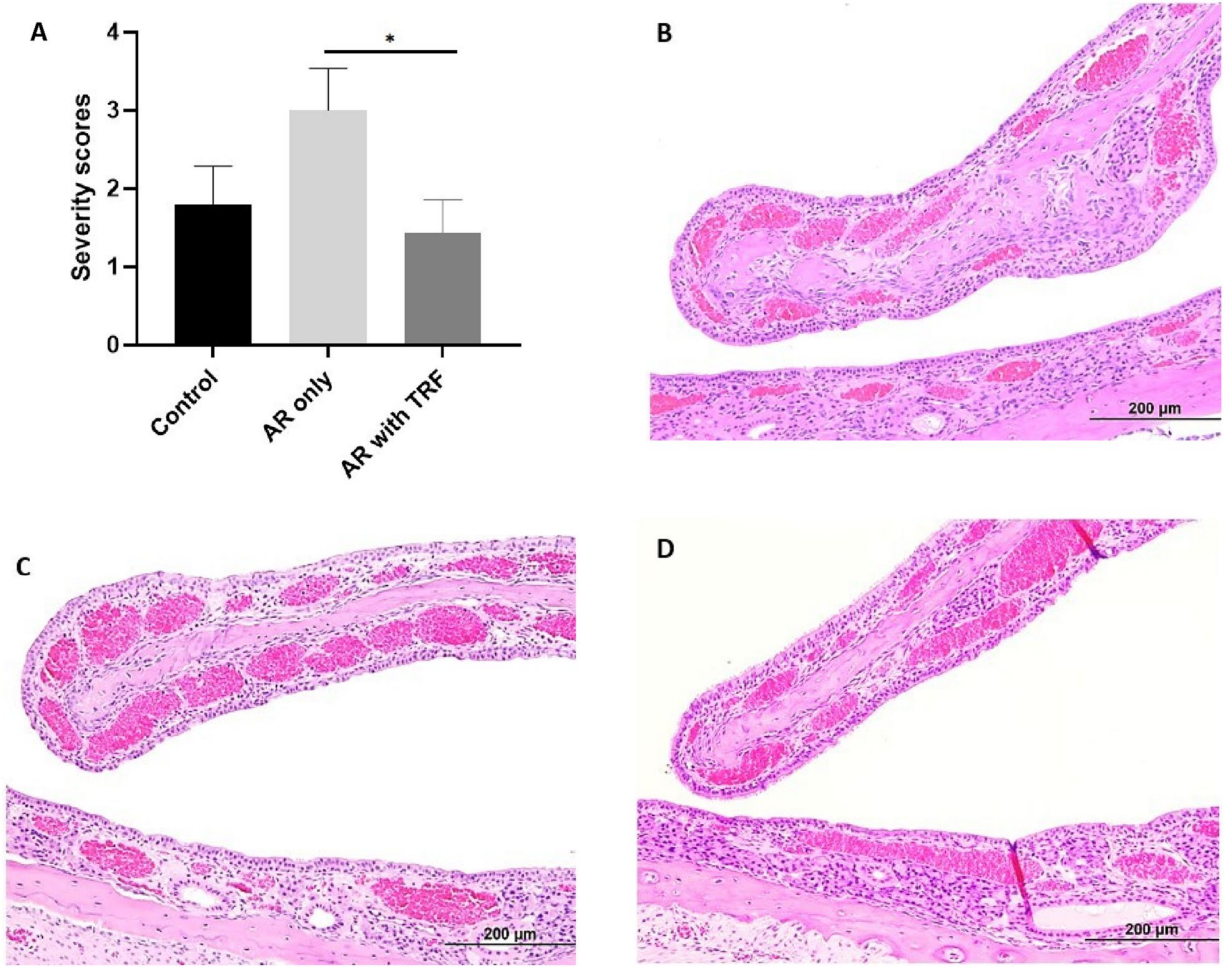
The severity scores of vascular congestion in submucosa (**A**) and light microscopic images of H&E staining in nasal section (**B**–**D**; ×200 magnification). (**B**) Control group shows minimal to mild degree of vascular congestion. (**C**) PAF-induced AR groups shows moderate degree of vascular congestion. (**D**) PAF-induced AR with TRF treatment group shows minimal degree of vascular congestion. The subjective scoring represents 0: no significant abnormalities; 1: minimal; 2: mild; 3: moderate; 4: marked; 5: severe. Data are representative of mean ± standard error (n = 19); *p < 0.05. AR, Allergic rhinitis; H&E, Hematoxylin and eosin; PAF, Platelet-activating factor; TRF, Tocotrienol-rich fraction

The histological evaluation of the nasal cavity, nasal septum and nasal turbinate including degree of inflammation, inflammatory cells infiltrate and mucosa damage in all groups are presented in Fig. 3. The severity of vascular congestion in submucosa is shown in Fig. 4.

#### Control group

In the control group, none to minimal degree of inflammation, polymorphonuclear cells infiltration and mucosa damage were observed (Fig. 3A). H&E staining demonstrated intact epithelial and regular goblet cells in the nasal septum and turbinate (Fig. 3B). Results for severity score (Fig. 4A) and H&E staining (Fig. 4B) showed the presence of mild vascular congestion in the submucosa. This is most likely a background change within normal limits.

#### PAF-induced AR group

Compared to the control group, there was a greater degree of inflammation and significant submucosal infiltration of polymorphonuclear cells in the PAF-induced AR group, with p < 0.05. An extensive damage in the mucosa was significantly observed as compared to the control and TRF treated group, with p < 0.0001 (Fig. 3A). H&E staining demonstrated aggregates of polymorphonuclear cells as well as the deprivation of epithelial and goblet cells due to substantial mucosa damage (Fig. 3C). Moreover, vascular congestion was significantly exacerbated by the PAF in AR rats (Fig. 4A and C).

#### PAF-induced AR group with TRF treatment

The overall histological parameters in the PAF-induced AR group receiving TRF treatment were restored to the regular nasal structures close to the healthy control, particularly in mucosa damage with p < 0.0001 (Fig. 3A). There was a marked improvement in the histological changes demonstrated by the H&E staining. A lower degree of inflammation, minimal infiltration of polymorphonuclear cells and minimal degree of mucosa damage were observed in the nasal structure (Fig. 3D). Furthermore, TRF significantly improved the vascular congestion when compared to PAF-induced AR group, with p < 0.05 (Fig. 4A and D).

## Discussion

Oxidative stress (OS) and inflammation are intricately linked where OS is intensified upon triggering inflammation [32]. The imbalance of antioxidants and free radicals such as reactive oxygen species (ROS) has been suggested to play a vital role in the pathogenesis of allergic diseases such as asthma, wheezing and atopic dermatitis, but little focus was given to Allergic rhinitis (AR) [33–36]. With the concept of ‘one airway, one disease’, it is likely that OS and inflammation take part in AR considering the upper and lower airways often share common disease associations and immunopathological mechanisms [37].

Current evidence suggests the involvement of PAF in the generation of ROS and acts as a powerful stimulator of acute inflammatory processes in allergic reactions [13, 38]. A rat model of PAF-induced rhinosinusitis has been successfully established by Jeon et al. where the presence of neutrophil clusters in nasal cavity, infiltration of polymorphonuclear cells in mucosa, goblet cell hyperplasia in the epithelium and epithelial damage were observed after one-time intranasal administration of 16 μg/ml PAF at 50 µl [39]. Besides, several human studies involving AR patients have proved the potency of PAF in inducing rhinitis like symptoms including rhinorrhoea and nasal congestion, as well as increasing eosinophilic and neutrophilic infiltration. PAF was also found in AR patients’ nasal secretions after antigen challenge of upper respiratory airway [40]. Moreover, the involvement of PAF in inducing oxidative bursts, priming polymorphonuclear cells for production and releasing of ROS was demonstrated [41, 42]. In line with these, Kato et al. reported a significant cellular adhesion and O_2_- release in PAF-induced human eosinophils [43]. All things considered, the engagement of PAF in allergic inflammation of airways in humans is plausible. In the current study, the PAF-induced AR model has been adopted to elucidate the role of PAF in the pathogenesis of AR. PAF has exacerbated AR symptoms in the AR group, especially in the infiltration of polymorphonuclear cells, mucosa damage and vascular congestion. Although ovalbumin (OVA) is one of the most widely used inducers for establishment of allergic respiratory in animal models, it acts as a multisystemic inducer and is administered in the presence of an adjuvant, with symptoms peaking after a period of time [44]. Unlike OVA, PAF induced nasal challenge can be done in a single dose with the appearance of the symptoms locally [45].

There is now increasing evidence for the immunological roles of platelets in inflammation, particularly in the development of allergic responses like asthma. Allergic asthma is a chronic inflammatory disease leading to mucus hypersecretion, as well as the hyperresponsiveness, obstruction and remodelling in the airways. Upon allergen exposure, platelets entail in the IgE-FcεRI-dependent pathway, resulted in the infiltration of central effector cells in allergic inflammation, and the release of platelets-derived factors such as type 2 cytokines and various biochemical mediators including PAF [46, 47]. ROS triggered by platelets also represent one of the contributing factors to allergic asthma. Considering AR and allergic asthma commonly coexist and appear to share key elements at the epidemiologic and pathophysiologic level, it could be speculated that platelets may also contribute directly to allergic inflammation in AR [48–50].

In the present study, we demonstrated the protective actions of TRF in ameliorating AR symptoms in a PAF-induced AR rat model. The dosage of TRF in the range of 5 to 16 μg/μl delivered at 10 μl was examined, with reference to the gold standard treatment of using nasal corticosteroids. Mometasone furoate (MF) as an intranasal aqueous spray is among the most common prescribed corticosteroids for AR. Its efficacy has been evaluated in several randomized, double-blind, placebo-controlled clinical trials involving adults and children with AR. Overall clinical data show MF aqueous spray, administered once daily at 100 μg per nostril for 2–12 weeks, is not only effective in treating AR, but also prevents the onset of symptoms in AR patients [51, 52]. In our work, TRF at highest dose of 160 μg markedly restored the nasal structures of the PAF-induced AR rats to normal as the healthy rats. Results from this study suggested TRF, as a natural phytonutrient, may provide symptomatic reliefs that is comparable to MF in the management of AR.

T3 possesses both antioxidant and anti-inflammatory properties. It not only can neutralize free radicals, but also has an anti-inflammatory action in allergic disorders [53, 54]. In a house dust-mite asthma-induced mouse model, Peh et al. demonstrated 250 mg/kg body weight of palm extracted γ-T3 given via oral gavage acts as a direct free radical scavenger by preventing airway inflammation and OS effectively as compared to α-TP. In addition, γ-T3 was able to inhibit the NF-κB nuclear translocation and promoted the levels of nuclear factor erythroid 2-related factor 2 (Nrf2). The results indicated the therapeutic potential of T3 for the treatment of allergic diseases [55]. In an OVA challenged allergic asthma model using Brown Norway rats, Zainal et al. reported oral supplementation of palm-derived TRF at 30 mg/kg body weight modulated the inflammatory reaction in bronchial asthma through the inhibition of Th2-derived proinflammatory cytokines production [56]. In another study, Wagner et al. described the therapeutic effects of dietary γ-TP supplementation in a rodent model of ozone-enhanced allergic nasal responses. Rats treated with oral γ-TP at 100 mg/kg body weight showed a decrease in the intraepithelial mucosubstances, eosinophilic inflammation and mucus-stimulating factors such as cysteinyl leukotrienes. This findings suggested the ability of TP in attenuating the effects of ozone in allergic upper airways of AR rat models [57]. Albeit TP was employed as the treatment agent in Wagner’s study, the hypothesis that T3, which possess 40–60 times stronger antioxidant and anti-inflammatory properties than TP [58], could provide protection against AR via similar mechanisms is plausible.

Nutritional epigenetics has established a new framework for gene-diet interactions by studying heritable modifications in gene expression without alterations in the underlying DNA sequence. The best-known epigenetic mechanism, DNA methylation, has been proposed to drive immune programming associated with pro-allergic in the epigenetic regulation of asthma and allergic diseases at the Th2 locus control region [59]. Vitamins and methyl donor nutrients were found to influence epigenetic programming on individual health throughout life by either inhibiting the enzyme involved or altering the availability of substrates required for those enzymatic activities. Several early life nutrition studies have shown a correlation between maternal dietary nutrients like folate and vitamin D and epigenetic phenomena in offspring that affect immune system function [60, 61]. Vitamin E in turn has antioxidative properties that affect epigenetic regulation of inflammation and DNA repair. Remely et al. observed a greater methylation pattern in the promoter region of the DNA repair gene MLH1, as well as a reduction in the DNA damage, in animals given vitamin E. Their findings support vitamin E's role as an epigenetic nutrient [62]. In a randomized, double-blind, placebo-controlled study, α-TP was found to enhance miR-9–3 promoter region methylation levels in obese and overweight women following 8 weeks of daily supplementation. The methylation of miR-9-3 led to miR-9 transcription, which is necessary for insulin secretion balance and glucose homeostasis, as shown by improved glycated haemoglobin profiles [63]. Taken together, these findings suggest vitamin E may affect the physiologic and pathologic processes of AR via epigenetic mechanisms, resulting in gene expression alterations.

At the moment, there is a lack of intervention human trial into the management of AR using T3. Nevertheless, the relationship between dietary vitamin E and allergic diseases in humans has been previously reported. Vitamin E supplementation was shown to protect young children from the development of atopy and wheezing [64]. Furthermore, patients with seasonal AR who received supplemental vitamin E supplementation in addition to their regular medication experienced an improvement in nasal symptom scores [65]. In another cross-sectional study, 65 children with AR and 48 healthy controls aged 6–14 years were assessed for the association between serum level of vitamin E and the occurrence and severity of AR [66]. The results revealed that children with AR had considerably lower serum vitamin E levels than normal children, and a negative correlation was found between level of vitamin E in serum and specific IgE along with skin prick test grade. Several investigations have also found a decrease in vitamin E and C levels, as well as an increase in oxidized glutathione in the airway lining fluids of allergic airway disease models [67, 68]. On the other hand, vitamin E does not appear to result in significant difference for nasal symptom severity or serum concentrations of specific IgE to common allergens in patients with perennial AR after 4 weeks of oral supplementation. The negative outcome of this study could be attributable to several factors. The synthetic form of vitamin E supplement examined, for example, may be less effective in terms of bioavailability and efficacy than the natural foam. Furthermore, vitamin E absorption may be suboptimal due to the uncontrolled amount and content of lipids consumed during supplementation. Moreover, some gastrointestinal and metabolic disorders such as intestinal malabsorption and cholestasis may affect vitamin E's effects [69]. In our study, intranasal TRF therapy offers the advantage of bypassing first-pass metabolism and facilitating TRF absorption by passive diffusion across the respiratory epithelium directly. In addition, high local levels of vitamin E in the nasal mucosa may help to scavenge ROS produced during the inflammatory process and repair the resulting epithelial damage in AR.

There may be some limitations to this study. More molecular research into signaling pathways such as phosphatidylinositol 3-kinase/protein kinase B (PI3K/Akt) and nuclear factor erythroid 2–related factor 2/heme oxygenase-1 (Nrf2/HO-1) would be beneficial in validating the potential mechanism of T3's antioxidant and anti-allergic effects. Despite significant anatomical similarities between humans and animals, the physiological and genetic variations may preclude dosage used in animal studies from being fully replicated in human trials. The inter-species dose extrapolation by using allometric scaling should be explored to determine a safer dose of PAF in humans. Finally, further randomized controlled clinical trials are needed to determine the therapeutic effects of TRF in the treatment of AR.

## Conclusion

TRF exhibited symptomatic relief actions in AR potentially due to its antioxidant, anti-inflammatory and anti-allergic properties. This is the first study to demonstrate the significance of intranasally administered TRF, which may offer an alternative therapeutic option in the prevention and reversal of AR.

## Data Availability

The data analysed and used in this study are available from the corresponding authors upon reasonable request.

## Abbreviations

α-T3: Alpha-tocotrienol
α-TP: Alpha-tocopherol
γ-T3: Gamma-tocotrienol
ANOVA: Analysis of variance
AR: Allergic rhinitis
ARIA: Allergic rhinitis and its impact on asthma
BSA: Bovine serum albumin
FcεRI: Fc epsilon RI
H&E: Hematoxylin and eosin
IgE: Immunoglobulin E
MF: Mometasone furoate
NF-κB: Nuclear factor kappa-light-chain-enhancer of activated B cells
Nrf2: Nuclear factor erythroid 2-related factor 2
Nrf2/HO-1: Nuclear factor erythroid 2-related factor 2/heme oxygenase-1
OS: Oxidative stress
OVA: Ovalbumin
PAF: Platelet-activating factor
PI3K/Akt: Phosphatidylinositol 3-kinase/protein kinase B
ROS: Reactive oxygen species
STAT 3: Signal transducer and activator of transcription 3
T3: Tocotrienol
TP: Tocopherol
TRF: Tocotrienol-rich fraction
